# Role of composite objective nutritional indexes in patients with chronic kidney disease

**DOI:** 10.3389/fnut.2024.1349876

**Published:** 2024-04-18

**Authors:** Bixia Yang, Yan Yang, Bicheng Liu, Min Yang

**Affiliations:** ^1^Department of Nephrology, The Third Affiliated Hospital of Soochwow University, Changzhou, China; ^2^Institute of Nephrology, Zhongda Hospital, Southeast University School of Medicine, Nanjing, China

**Keywords:** geriatric nutritional risk index, prognostic nutritional index, controlling nutritional status score, chronic kidney disease, composite objective nutritional indexes

## Abstract

Malnutrition persists as one of the most severe symptoms in patients with chronic kidney disease (CKD) globally. It is a critical risk factor for cardiovascular and all-cause mortality in patients with CKD. Readily available objective indicators are used to calculate composite objective nutritional assessment indexes, including the geriatric nutritional risk index, prognostic nutritional index, and controlling nutritional status score. These indexes offer a straightforward and effective method for evaluating nutritional status and predicting clinical outcomes in patients with CKD. This review presents supporting evidence on the significance of composite nutritional indexes.

## 1 Introduction

Patients with chronic kidney disease (CKD), especially those with end-stage renal disease (ESRD), are highly susceptible to malnutrition (1), resulting in a decreased quality of life (2). Patients with CKD face increased risks of worsening their prognosis owing to nutrition-related issues, including cachexia and protein-energy wasting (PEW). In addition, it is associated with increased mortality rates (3, 4). Nutritional intervention has been demonstrated to enhance the long-term nutritional status and survival rate of patients with CKD (5). Consequently, there is a crucial need for reliable nutritional evaluation indicators to assist clinicians in the early identification of malnutrition and implementation of measures to enhance prognosis.

## 2 Relationship between malnutrition, inflammation, cardiovascular disease, and mortality in chronic kidney disease

Patients with CKD exhibit a higher prevalence of cardiovascular disease (CVD) and increased mortality than those without CKD (6). In a study by Okabe et al., the influence of nutritional conditions on coronary atherosclerosis was investigated in patients undergoing hemodialysis (HD) using optical coherence tomography (7). Their findings revealed that malnourished patients exhibited larger calcified plaques in affected areas at baseline and demonstrated a more significant change in calcified plaque angle in unaffected areas after 6 months. Malnutrition may contribute to the progression of coronary artery calcification in patients undergoing HD.

Furthermore, the malnutrition-inflammation complex syndrome indicates a close relationship between malnutrition and inflammation in patients undergoing dialysis (8). In addition, malnutrition-inflammation atherosclerosis underscores the significance of atherosclerotic diseases arising from malnutrition and inflammation (9). Malnutrition and chronic inflammation exhibit a mutual causal relationship. Malnourished patients are highly vulnerable to infection and chronic inflammation due to their decreased immunity (10). However, proinflammatory cytokines contribute to an increased prevalence of chronic fatigue and the breakdown of muscle proteins, resulting in muscle atrophy and malnutrition (11). In addition, inflammation elevates resting energy expenditure in patients with CKD (12). Comorbid conditions are another prevalent factor contributing to protein-energy malnutrition and inflammation (8). In summary, malnutrition and inflammation are inherently interconnected in patients with CKD. In addition, Liu et al. demonstrated a significant association between hypersensitive C-reactive protein (hs-CRP) levels and CVD events in patients with CKD. Compared with various indices related to CVDs, hs-CRP exhibited the most potent predictive effect for CVD events (13). Moreover, a substantial cohort study included 2,399 participants with CKD without a history of CVD at study entry. The study demonstrated that inflammation biomarkers, including IL-6 and TNF-a, are independently associated with CVD events and mortality in patients with CKD (14).

In summary, malnutrition, either directly or via chronic inflammation, results in atherosclerosis, which subsequently increases cardiovascular (CV) events and mortality. Therefore, a timely and precise assessment of nutritional status is crucial for the prognosis of patients with CKD.

## 3 Available nutritional assessment methods for patients with chronic kidney disease

Nutritional status can be evaluated using various screening/diagnostic approaches in addition to physical examinations and evaluations of nutritional intake. These approaches can be either subjective or objective.

### 3.1 Subjective nutritional screening tools

The first fundamental approaches for evaluating nutritional status were subjective nutritional screening tools. To date, there are five established subjective nutritional screening tools: Subjective Global Assessment (SGA), Malnutrition Inflammation Score (MIS), Mini Nutritional Assessment Short Form, Nutritional Risk Score, and Malnutrition Universal Screening Tool.

The SGA was thoroughly assessed considering factors such as dietary intake, weight change, specific symptoms (including gastrointestinal symptoms), and physical examination. It has gained widespread recognition as a valuable clinical nutrition evaluation tool. The Kidney Disease Outcomes Quality Initiative guideline specifically recommends using SGA for nutritional evaluation in patients with ESRD (15). MIS, derived from SGA, incorporates body mass index (BMI), total iron binding capacity, and serum albumin to evaluate nutritional status and inflammatory response (16). Nonetheless, these subjective nutritional screening tools are primarily assessed by professional clinicians, and potential biases may arise from differences in clinician perspectives.

### 3.2 Anthropometric methods and body composition assessment

In addition to subjective nutritional assessment indicators, objective approaches are widely employed and are convenient in clinical practice. Human measurement approaches, including weight, BMI, and upper arm circumference, cannot differentiate between body components. Furthermore, fluid imbalance in patients with CKD can influence nutritional assessment (17). Therefore, when employing body weight evaluation, careful attention should be given to changes in body weight, particularly when edema is caused by various factors depending on the condition of the patient.

Furthermore, the evaluation of body composition is an attractive method. Bioelectrical impedance analysis includes parameters such as muscle tissue index, adipose tissue index, muscle tissue content, adipose tissue content, dry weight, edema index, phase angle, and volume load. Research has demonstrated a correlation between muscle mass index and adipose tissue index with nutritional status in nondialysis patients with stage 3 to 5 CKD (18). Nevertheless, its application in patients on dialysis poses challenges due to fluid balance fluctuations. In addition, it is a more costly method than other approaches.

### 3.3 Laboratory measurements

It is essential to highlight laboratory measurements relevant to nutrition evaluation, primarily albumin, pre-albumin, and cholesterol levels. Serum albumin plays a protective role in renal function. Albumin can mitigate the nephrotoxicity of interleukin-2 (IL-2). Increased serum albumin concentration is advantageous for the survival of cultured renal tubular cells (19). Albumin safeguards the kidney by scavenging reactive oxygen species, preventing oxidative damage, and binding and transporting lysophosphatidic acid-protective acids (20). In addition, albumin enhances renal perfusion by reacting with nitrogen oxides or binding to platelet-activating factors, thereby prolonging effective renal vasodilation (21). Albumin can also stimulate DNA synthesis in renal tubular cells through signaling pathways that involve Ca^2+^, protein kinase C, epidermal growth factor receptor, mitogen-activated protein kinase, and nuclear factor-κB (22, 23). Although serum albumin serves as an indicator of nutritional status, it also, to a certain extent, functions as a marker of overhydration and inflammation (24). Thus, it alone cannot provide an accurate evaluation of nutritional status. Pre-albumin is a superior index of nutritional status, possessing greater sensitivity than albumin due to its lower content and short half-life in serum, making it more responsive to acute changes in proteins (25). A prospective cohort study revealed that pre-albumin levels were a predictive factor of 3-year mortality and hospitalization in patients with CKD. However, similar to albumin, pre-albumin, may be influenced by the inflammatory state of the patient. Therefore, it is not routinely examined as a laboratory parameter in patients with CKD (26). Furthermore, cholesterol serves as another laboratory test indicator to assess nutritional status. It is an undeniable risk factor for CVD and mortality because it accurately reflects the patient’s lipid metabolism. The kidney plays a crucial role in the synthesis, transport, efflux, and breakdown of cholesterol (27). Cholesterol metabolism disorders within the visceral layer of the renal utricle cells, endothelial cells, and renal tubular cells can result in cholesterol accumulation. This accumulation can induce inflammation and oxidative stress, ultimately resulting in renal dysfunction and failure (28). Furthermore, the accumulation of fatty acids, a metabolite of cholesterol, can trigger mitochondrial and renal cell damage by activating the innate immune system and promoting inflammation that contributes to fibrosis. Nonetheless, it appears to be a protective characteristic that is associated with increased survival in dialysis patients. This phenomenon is called reverse epidemiology (29). However, it has the drawback of not reflecting the condition of other body components, such as proteins, in patients.

### 3.4 Composite objective nutritional indexes

In summary, the nutritional evaluation approaches discussed above, whether subjective or objective, have inherent limitations and cannot provide a comprehensive assessment of the nutritional status of patients with CKD. In recent years, various composite objective nutritional evaluation indexes, including the geriatric nutritional risk index (GNRI), prognostic nutritional index (PNI), and controlling nutritional status (CONUT) score, have proven effective in evaluating the nutritional condition and predicting clinical prognosis in patients with CKD, regardless of whether they are undergoing HD, peritoneal dialysis (PD), or not (30–33). These composite indicators are computed using several objective indicators, which provide a comprehensive evaluation of the patient’s nutritional status. They have found extensive applications in various diseases, including CKD.

#### 3.4.1 Geriatric nutritional risk index

Originally established to predict the risk of adverse outcomes in senior inpatients (34), the GNRI is computed as follows: GNRI = 14.89 × serum albumin (g/dL) + [41.7 × actual body weight/ideal body weight]. Ideal body weight is determined from body height and gender using the following formulas: ideal body weight for males = body height − 100 − [(body height − 150)/4], and ideal body weight for females = body height − 100 − [(body height − 150)/ 2.5]. If the ratio of actual body weight to ideal body weight exceeds 1, it is set to 1 (29). Nonetheless, in some studies, the ideal body weight was calculated based on height and a BMI of 22 kg/m2. The GNRI scores obtained from both formulas exhibited minimal differences (35).

Current evidence indicates that GNRI is useful in patients with cancer, heart failure, respiratory failure, and stroke (36–39). Numerous studies have shown that GNRI is effective in assessing the nutritional status of patients with CKD, irrespective of whether they are undergoing HD, PD, or non-dialysis. In a study by Yamada et al., GNRI and MIS exhibited the highest accuracy compared with various other nutritional screening measures in HD patients (35). In a recent prospective observational study involving 318 patients undergoing maintenance HD, GNRI exhibited higher sensitivity and specificity, whereas MIS demonstrated superior performance in some etiologic and phenotypic components (40). Furthermore, Ren et al. demonstrated that GNRI serves as a convenient and practical index for assessing the nutritional status of patients undergoing PD (4). Consequently, GNRI has gained increasing recognition as a valuable nutritional evaluation index for patients with CKD.

In addition to its function as a nutritional evaluation tool, numerous researchers have explored the predictive value of GNRI in the prognosis of patients with CKD. Kobayashi et al. were the pioneers in demonstrating that GNRI can predict mortality in patients undergoing HD (41). A large prospective study conducted in Japan indicated that lower GNRI levels were associated with increased infection-related mortality in patients undergoing HD, particularly in older patients (42). Another Japanese study involving 3,536 HD patients reported that GNRI performed well in predicting all-cause mortality (43). Consistent findings have been substantiated in other investigations conducted in China, Italy, and Korea (3, 44–46). Furthermore, a meta-analysis provided additional support, indicating that GNRI can be employed to identify patients at high risk of death in patients undergoing HD (47). In addition, the predictive role of GNRI for mortality was demonstrated in peritoneal and non-dialysis patients. Ren et al. established a strong correlation between GNRI and mortality in patients undergoing PD, whereas a multicenter prospective cohort study involving Chinese patients with CKD indicated that GNRI had a certain predictive value for the prognosis of non-dialysis patients (4, 48). In a recent Korean population-based study including 34,933 patients, the best GNRI cutoff value for predicting death among patients undergoing maintenance HD was 90.8 (3). Several studies have explored the predictive value of GNRI for all-cause mortality and CV mortality in patients with CKD (Table 1).

**Table 1:** Summary of cohort studies investigating the relationship of geriatric nutritional risk index with outcomes in patients with chronic kidney disease (CKD).

Patients	Authors, year	Country	Age (Year)	N, Male (%)	Follow-up time	Cutoff value of GNRI	Outcomes	Ref.
HD	Kobayashi et al., 2010	Japan	60 ± 12	490, 59.8%	60 months	90	All-cause mortality	(41)
	Panichi et al., 2014	Italy	65.7 ± 14.1	753, 60.7%	84 months	92	All-cause mortality	(45)
	Takahashi et al., 2014	Japan	64 ± 13	1,568, 66.9%	63 months	92	All-cause and CV mortality	(49)
	Nakagawa et al., 2015	Japan	59.8 ± 10.2	133, 57.9%	72 months	96	All-cause and CV mortality	(50)
	Komatsu et al., 2015	Japan	65.4 ± 13.2	332, 64.2%	36 months	92	All-cause mortality	(51)
	Ishii et al., 2017	Japan	64 ± 12	973, 62.6%	96 months	91.2	All-cause and CV mortality	(52)
	Takahashi et al., 2017	Japan	64 ± 11	409, 55.3%	52 months	92.2	All-cause mortality	(53)
	Chen et al.,2019	China	53.9 ± 15.1	1,025, 57.4%	28.1 months	92	All-cause and CV mortality	(44)
	Matsukuma et al., 2019	Japan	63.7 ± 12.2	3,436, 59.1%	48 months	95.8	All-cause and infection-related mortality	(42)
	Yamada et al., 2020	Japan	66 (58–74)*	3,536, 65%	26 months	89.3	All-cause mortality	(43)
	Mizuiri et al., 2021	Japan	67 (56–74)#	264, 65.2%	24 months	92	All-cause mortality	(54)
	Yajima et al., 2021	Japan	63.4 ± 13.9	180, 68.3%	55.2 months	91.2	All-cause and CV mortality	(55)
	Fujioka et al., 2022	Japan	68.3 ± 12.4	183, 53.6%	66 months	91.6	All-cause and CV mortality	(56)
	Yajima et al., 2022	Japan	63.8 ± 13.7	263, 66.5%	37 months	91.2	All-cause mortality	(57)
	Wang et al., 2023	China	63.3 ± 13.6	240, 74%	58 months	89.0	All-cause and CV mortality	(46)
	Kim et al., 2023	Korea	60.2 ± 13.0	34,933, 58.8%	53.7 months	90.8	All-cause mortality	(3)
PD	Kang et al., 2013	Korea	52.5 ± 15.1	486, 53.1%	36 months	96.4	All-cause mortality	(58)
	Lee et al., 2017	Korea	50.8 ± 11.9	133, 51.9%	51.1 months	96.7	CV events	(59)
	Ren et al., 2020	China	50.2 ± 14.4	1804, 55.4%	33.7 months	94.55	All-cause mortality	(4)
	Yıldırım et al., 2023	Turkey	52 (41–60)#	220, 48.6%	33.5 months	–	All-cause mortality	(60)
NDD-CKD	Maruyama et al., 2016	Japan	63.5 ± 9.2	161, 51%	96 months	96	CV events	(61)
	Kiuchi et al., 2016	Japan	67 (37–81)##	126, 51.6%	64 months	92	All-cause mortality and CV events	(62)
	Lin et al., 2019	Taiwan of China	66 ± 13	326, 68.7%	58.8 months	92.4	All-cause mortality and CV events and ESRD	(31)
	Xiong et al., 2020	China	48.6 (38.2–59.6)#	2,791, 58.9%	52.6%	86	All-cause mortality and CV events	(48)

CKD, chronic kidney disease; HD, hemodialysis; PD, peritoneal dialysis; CV, cardiovascular; GNRI, Geriatric nutritional risk index; NDD, non-dialysis-dependent; ESRD, end-stage renal disease; *median (95% CI); ^#^median (interquartile ranges). ^##^median (10th –90th percentile).

Not only the baseline GNRI but also the changes in GNRI can effectively evaluate nutritional status and predict mortality in patients with CKD. Cho et al. discovered that serial GNRI evaluation served as a valuable predictive factor for mortality in patients undergoing HD (63). Yıldırım et al. demonstrated that GNRI variability was independently associated with mortality in patients undergoing PD (60). Undoubtedly, monitoring changes in GNRI values can enhance the accuracy of predicting mortality.

GNRI is computed based on serum albumin, body height, and weight. BMI is calculated from height and weight. Serum albumin and BMI serve as indicators to assess the nutritional condition of patients with CKD. Furthermore, Cooper et al. demonstrated that hypoalbuminemia can predict vascular morbidity and mortality (64), and a meta-analysis affirmed that a higher BMI may be protective against all-cause mortality in populations undergoing HD (65). However, a large cohort study revealed that GNRI was a superior predictor of CVD mortality at the onset of HD treatment compared with serum albumin and BMI (49). Thus, GNRI performs well in evaluating nutritional status and predicting mortality risk in patients with CKD.

Furthermore, additional studies have indicated the value of GNRI in predicting muscular strength, fractures, brain infarction, and hemorrhage in patients undergoing maintenance HD (66–68).

In summary, GNRI has found extensive applications in CKD, particularly in predicting CV and all-cause mortality. Further investigation is needed to understand the role of GNRI in predicting other outcomes in patients with CKD and to determine appropriate cutoff values.

#### 3.4.2 Prognostic nutritional index

PNI is an immune-nutritional index developed by Japanese scholars Onodera et al. to assess the nutritional and immunological status of surgical patients with gastrointestinal cancer (69). PNI was obtained from the lymphocyte count of the peripheral blood and serum albumin level. PNI was computed using the following formula: PNI = serum albumin (g/L) + 5 × lymphocyte count (10^9^/L). It serves as an index reflecting nutritional status, immune condition, and inflammation status. Recent studies have indicated that PNI serves as a novel predictor for the prognosis of various diseases, including solid tumors, hematologic tumors, heart failure, and COVID-19 (70–73).

Research on PNI has gained increasing attention in patients with CKD, particularly those undergoing dialysis. A multi-center study conducted in China revealed that a low PNI level was an independent risk factor for high peritoneal transport status in patients undergoing PD (74). Patients undergoing PD with a high peritoneal transport state face increased risks of mortality and technique failure (TF) (75). In addition, our study demonstrated associations between PNI and CONUT with mortality, CVD events, and TF in patients undergoing PD, indicating that they may serve as better predictors than GNRI (76, 77). In our study, the CONUT score exhibited the highest area under the curve (AUC; 0.733; 95% CI, 0.674–0.787) for predicting all-cause mortality, whereas the PNI exhibited the highest AUC for predicting CVD (0.718; 95% CI, 0.658–0.773) and TF (0.600, 95% CI 0.539–0.658). These results are consistent with the findings of Peng et al. in 2017 (78), indicating that a low PNI at the beginning of PD is independently associated with an increased risk of CVD mortality. Furthermore, patients undergoing PD have a poor immune status and are highly vulnerable to infection. A study involving 899 patients undergoing PD demonstrated that PNI could independently predict new-onset pneumonia (79).

In a recent study comprising 101,616 patients undergoing HD, PNI demonstrated superior predictive performance for mortality compared with serum albumin level or total lymphocyte count alone. It has emerged as a simple and practical predictor in patients undergoing HD (80). Considering that PNI was originally designed to evaluate the nutritional status of patients with cancer undergoing surgery, its effectiveness in evaluating nutritional status and predicting outcomes in HD patients undergoing surgery is a pertinent question. Kurumisawa et al. demonstrated that a PNI of ≤ 34 served as a robust independent predictive factor for long-term mortality in HD patients undergoing cardiac surgery (81). Enhanced nutrition based on preoperative PNI may enhance surgical outcomes in patients undergoing HD. The most crucial operation for HD in patients with ERSD is the arteriovenous fistula (AVF). To examine the predictive role of some markers in AVF maturation failure, Kaller et al. conducted a retrospective cohort study, and their findings indicated that higher preoperative PNI values can effectively predict AVF maturation failure, early thrombosis, and short-term mortality (82).

Furthermore, PNI can predict the prognosis of nondialysis patients. Barutcu et al. discovered a correlation between PNI and mortality in elderly patients with CKD (32). Thus, PNI can be employed to assess the nutritional status of these patients and potentially enhance outcomes. In addition, a study in China established that PNI independently predicted the progression of diabetic nephropathy (DN) in patients with type 2 diabetes (83). Therefore, PNI plays a crucial role in identifying high-risk patients with diabetes, thereby enabling personalized management and treatment. In addition, a study from Korea indicated that among various nutritional indices, PNI and CONUT scores exhibited a stronger relationship with disease activity, with the PNI score specifically correlated with ESRD in patients with lupus nephritis (84). Based on the aforementioned findings, in the future, our center plans to investigate the value of PNI in evaluating the nutritional status and prognosis of patients with various pathological types of CKD, including IgA nephropathy and hypertensive nephropathy. Regarding CV outcomes, only a cross-sectional investigation has demonstrated that PNI and CONUT are independently related to prior CVD in patients with CKD (85). A longitudinal study is needed to clarify the association between these two indices and future CV events.

In general, patients with CKD and coronary artery disease (CAD) face an increased risk of developing contrast-associated acute kidney injury (CA-AKI). Current guidelines recommend the use of the Global Registry of Acute Coronary Events (GRACE) score for predicting mortality and major adverse cardiovascular events (MACEs) in patients with acute coronary syndrome (ACS) (86). However, when the GRACE risk score is employed, the major events in dialysis patients are significantly underestimated (87). A study involving 4,391 patients revealed a negative correlation between PNI and CA-AKI in patients with CKD and CAD undergoing coronary angiography (88). Ni et al. discovered that a lower PNI was independently associated with an increased risk of mortality and enhanced the predictive capability of the GRACE score for overall death and MACEs in patients with ACS and CKD. Similar findings were observed with CONUT and PNI (89). Thus, enhancing the pre-angiography nutritional status of patients with CKD based on the PNI value can reduce the incidence of CA-AKI.

Table 2 summarizes the studies investigating the predictive value of PNI for outcomes in patients with CKD.

**Table 2:** Summary of cohort studies investigating the relationship of prognostic nutritional index with outcomes in patients with CKD.

Patients	Authors, year	Country	Age (Year)	N, Male (%)	Follow-up time	Cutoff value of PNI	Outcomes	Ref.
HD	Kurumisawa et al., 2018	Japan	66.5 ± 9	110, 74.5%	28 months	34	All-cause mortality after cardiac surgery	(81)
	Miyasato et al., 2023	USA	63 ± 15	101,616, 56%	16.8 months	39.5	All-cause mortality	(80)
PD	Peng et al., 2017	China	50.8 ± 14.5	345, 59.1%	25.3 months	36.6	All-cause and CV mortality	(78)
	Yang et al., 2020	China	43.8 ± 14.9	252, 58.3%	22.8 months	37.55	All-cause mortality and CVD events	(76)
	Yang et al., 2022	China	48.2 ± 14.6	276, 57.6%	30 months	40.2	Technique Failure	(77)
	Shang et al., 2022	China	59(47–67)#	899, 57.4%	41.4 months	36.1	The first occurrence of pneumonia	(79)
	Huang et al., 2023	China	51.1 ± 14.9	404, 58.66%	3–80 months	36.6	High peritoneal transport status	(74)
NDD-CKD	Kaller et al., 2022	Romania	61.1 ± 13.8	125, 60.8%	6 weeks	40.59	The six-week maturation rate, early thrombosis, and mortality	(82)
	Ahn et al., 2019	Korea	36(27–46)#	207, 10.1%	57.1 months	35.41	ESRD	(84)
	Dong et al., 2021	China	68.7 ± 10	4,391, 73.38%	–	37.9	CA-AKI	(88)
	Zhan et al., 2022	China	51.6 ± 8.8	321, 70.7%	30 months	–	ESRD	(83)
	Atas et al., 2022	Japan	85.7 ± 3.7	359, 49.3%	37.9 months	39	All-cause mortality	(32)
	Tsuda et al., 2023	Japan	67(56–76)#	2,751, 55%	–	46.47	Prior CVD	(85)
	Ni et al., 2023	China	73(65–80)#	705, 74.89%	31 months	38	All-cause mortality and MACEs	(89)

CKD, chronic kidney disease; HD, hemodialysis; PD, peritoneal dialysis; CV, cardiovascular; CVD, cardiovascular disease; PNI, Prognostic nutritional index; NDD, non-dialysis-dependent; ESRD, end-stage renal disease; MACE, major adverse cardiovascular events; ^#^median (interquartile ranges).

The pathophysiological mechanisms of albumin in CKD have been described above. In addition, lymphocytes play a crucial role in CKD. Various T cell subsets and innate lymphoid cells mediate kidney injury and fibrosis by modulating endogenous cell death, proliferation, and fibroblast formation. In this process, cytokine secretion and cytotoxicity are the two primary mechanisms (90). Some lymphocyte subsets, including Th1, Th17, type I NKT cells, and γδT cells, generate pathogenic cytokines such as IFN-γ, inducing nephritis and causing damage to renal tubular epithelial cells (TEC) and vascular endothelial cells, ultimately resulting in decreased renal function and aggravated renal fibrosis. Conversely, mucosa-associated invariant T cells play a pathogenic role through cytotoxicity. Additional subtypes, including Tregs, DNT cells, ILC2, and type II NKT cells, generate protective cytokines such as IL-10, which mitigate renal inflammation and facilitate TEC repair, thereby protecting renal function and mitigating renal fibrosis. Furthermore, Th2 and CD8 T cells exhibit dual roles in CKD. Consequently, in a non-inflammatory state, lymphocyte counts serve, to some extent, as indicators of the immune status of patients with CKD.

#### 3.4.3 Controlling nutritional status score

The CONUT score was initially developed for the early evaluation of nutritional status among hospitalized patients. This score comprises serum albumin, total cholesterol level, and total lymphocyte count with certain weights (Tables 2, 3) (91). These three indicators primarily signify protein storage, immune status, and lipid metabolism in patients. In addition, the CONUT score offers a comprehensive assessment of nutrition, and these three components are routine laboratory tests conducted during hospitalization and are easy to obtain. The CONUT score categorizes nutritional status into normal, light, moderate, and severe. As previously mentioned, higher values of GNRI and PNI indicate better nutritional status and prognosis, whereas the CONUT score shows the opposite trend. Several researchers have investigated the predictive role of the CONUT score in the outcomes of patients with heart and brain diseases, as well as tumors affecting the digestive system, respiratory system, and blood system (92–96).

In recent years, the relevance of the CONUT score in CKD has attracted significant attention. As mentioned above, similar to PNI, the CONUT score enhanced the predictive ability of the GRACE score for overall death and MACEs in patients with ACS and CKD (89). In addition, the CONUT score exhibited an independent relationship with prior CVD in patients with CKD and demonstrated a superior correlation with disease activity in patients with lupus nephritis compared with other nutritional evaluation indexes (84, 85). However, no study has explored the longitudinal prediction of the CONUT score for CVD outcomes in non-dialysis patients with CKD or its impact on progression in patients with lupus nephritis. Meanwhile, Huo et al. emphasized that the CONUT score was a significant risk factor for ESRD, CVD events, and all-cause mortality in patients with DN (97). Their findings are consistent with the results of Zhang et al., who indicated that malnutrition has predictive value for the progression of DN (83).

Regarding dialysis, Takagi et al. discovered that the CONUT score demonstrated predictive capabilities for all-cause mortality and infectious disease mortality, and its predictive value was higher in patients with CKD who had just started dialysis (98). In addition, their study revealed that only 8.7% of patients on dialysis were considered nutritionally normal. In summary, PEW is prevalent in patients undergoing dialysis. In addition, our center conducted a retrospective and observational study to elucidate the significance of the CONUT score at the beginning of PD (33, 76, 77). The findings revealed that approximately 74% of the patients exhibited high CONUT scores (CONUT score > 3), indicating malnutrition. In contrast, a previous study found that 42% of patients undergoing PD were diagnosed as malnourished using SGA during initial dialysis (99). Meanwhile, the CONUT score was confirmed to be a reliable predictor of overall death, CVD, and TF. Furthermore, in another study, the predictive value of PD-associated peritonitis was demonstrated for the CONUT score and dialysis age, with a higher predictive value observed for the combined diagnosis (100). Unlike previous studies, our study examined the baseline and half-year CONUT scores instead of solely relying on a single baseline CONUT score. It is essential to recognize that our study has some limitations. Consequently, it is recommended that nutritional and immune support before treatment be assessed based on the CONUT score in prospective, randomized controlled studies.

**Table 3:** Evaluation of the controlling nutritional status (CONUT) score.

Parameter	CONUT			
	Normal	Light	Moderate	Severe
Serum albumin (g/dl)	≥3.5	3.0–3.4	2.5–2.9	<2.5
Albumin score	0	2	4	6
Total lymphocytes (count/mm^3^)	≥1,600	1,200–1,599	800–1,199	<800
Lymphocytes score	0	1	2	3
Total cholesterol (mg/dl)	>180	140–180	100–139	<100
Cholesterol score	0	1	2	3
CONUT score(total)	0–1	2–4	5–8	9–12
Assessment	Normal	Light	Moderate	Severe

Table 4 summarizes the studies investigating the predictive role of the CONUT score for outcomes in patients with CKD.

**Table 4:** Summary of cohort studies investigating the relationship between CONUT score and outcomes in patients with CKD.

Patients	Authors, year	Country	Age (Year)	N, Male (%)	Follow-up time	Cutoff value of CONUT	Outcomes	Ref.
Dialysis	Takagi et al., 2022	Japan	69 ± 12	311, 73%	37 months	–	All-cause death and death from CVDs and infectious diseases	(98)
PD	Zhou et al., 2020	China	48.3 ± 14.9	252, 58.3%	22.8 months	3	All-cause mortality, CVD and technique failure	(33)
	Yang et al., 2020	China	43.8 ± 14.9	252, 58.3%	22.8 months	3	All-cause mortality and CVD events	(76)
	Yang et al., 2022	China	48.2 ± 14.6	276, 57.6%	30 months	3	Technique Failure	(77)
	Wu et al., 2023	China	48 (37–60)#	324, 58%	33 months	-	Peritoneal dialysis-associated peritonitis	(100)
NDD-CKD	Ahn et al., 2019	Korea	36 (27–46)#	207, 10.1%	57.1 months	–	ESRD	(84)
	Tsuda et al., 2023	Japan	67 (56,76)#	2,751, 55%	–	1	Prior CVD	(85)
	Huo et al., 2023	China	50.5 (45–59)#	336, 64.9%	61 months	3	ESRD, CVD, and All-cause mortality	(97)
	Ni et al., 2023	China	73 (65–80)#	705, 74.89%	31 months	–	All-cause mortality and MACEs	(89)

CKD, chronic kidney disease; HD, hemodialysis; PD, peritoneal dialysis; CV, cardiovascular; CVD, cardiovascular disease; CONUT, Controlling nutritional status; NDD, non-dialysis-dependent; ESRD, end-stage renal disease; ^#^median [interquartile ranges (IQR)].

## 4 Differences between the geriatric nutritional risk index, prognostic nutritional index, and controlling nutritional status score

When considering composition, serum albumin is a common component of these three composite objective nutritional indexes. On this basis, GNRI considers body weight, PNI incorporates blood lymphocyte count, and the CONUT score considers cholesterol alongside the two laboratory indexes included in PNI.

GNRI has been the most extensively investigated and comprehensive in patients with CKD compared with the other two composite objective nutritional indexes. GNRI has been examined both as a dichotomous and a continuous variable, whereas PNI and CONUT scores have only been investigated as dichotomous. GNRI has demonstrated diverse predictive clinical outcomes in patients with CKD compared with the other two composite objective nutrition indicators. Particularly, PNI can predict the surgical outcomes of patients with CKD. In addition, PNI and CONUT were correlated with the activity of lupus nephritis.

## 5 Conclusion

Patients with CKD often experience malnutrition, a condition that can independently increase CV events and CV mortality or contribute to these outcomes through inflammation mediation. Consequently, nutritional management plays a crucial role in providing high-quality care to this population. Composite objective nutritional indexes, including GNRI, PNI, and the CONUT score, are simple and effective nutrition evaluation indicators. This review highlights the valuable utility of composite objective nutritional indexes in evaluating the nutritional status and predicting clinical outcomes in patients with CKD.

This review also has certain limitations. Most of the studies on composite objective nutritional indexes in kidney disease were based on East Asian populations. Supporting evidence for other ethnic groups is still lacking. In addition, PNI and CONUT have been studied relatively rarely in dialysis patients, and more studies are needed to demonstrate the effectiveness of these two indicators in dialysis patients.

## Author contributions

BY: Methodology, Formal analysis, Writing – original draft. YY: Validation, Writing – review & editing, Funding acquisition. BL: Conceptualization, Supervision. MY: Project administration, Funding acquisition.
